# Neurochemical characterization of myenteric neurons in the juvenile gilthead sea bream (*Sparus aurata*) intestine

**DOI:** 10.1371/journal.pone.0201760

**Published:** 2018-08-03

**Authors:** Chiara Ceccotti, Cristina Giaroni, Michela Bistoletti, Manuela Viola, Francesca Crema, Genciana Terova

**Affiliations:** 1 Department of Biotechnology and Life Sciences, University of Insubria, Varese, Italy; 2 Department of Medicine and Surgery, University of Insubria, Varese, Italy; 3 Department of Internal Medicine and Therapeutics, Section of Pharmacology, University of Pavia, Pavia, Italy; 4 Inter-University Centre for Research in Protein Biotechnologies "The Protein Factory"- Polytechnic University of Milan and University of Insubria, Varese, Italy; Lewis Katz School of Medicine at Temple University, UNITED STATES

## Abstract

We evaluated the chemical coding of the myenteric plexus in the proximal and distal intestine of gilthead sea bream (*Sparus aurata*), which represents one of the most farmed fish in the Mediterranean area. The presence of nitric oxide (NO), acetylcholine (ACh), serotonin (5-HT), calcitonin-gene-related peptide (CGRP), substance P (SP) and vasoactive intestinal peptide (VIP) containing neurons, was investigated in intestinal whole mount preparations of the longitudinal muscle with attached the myenteric plexus (LMMP) by means of immunohistochemical fluorescence staining. The main excitatory and inhibitory neurochemicals identified in intestinal smooth muscle were ACh, SP, 5HT, and NO, VIP, CGRP. Some neurons displayed morphological features of ascending and descending interneurons and of putative sensory neurons. The expression of these pathways in the two intestinal regions is largely superimposable, although some differences emerged, which may be relevant to the morphological properties of each region. The most important variances are the higher neuronal density and soma size in the proximal intestine, which may depend on the volume of the target tissue. Since in the fish gut the submucosal plexus is less developed, myenteric neurons substantially innervate also the submucosal and epithelial layers, which display a major thickness and surface in the proximal intestine. In addition, myenteric neurons containing ACh and SP, which mainly represent excitatory motor neurons and interneurons innervating the smooth muscle were more numerous in the distal intestine, possibly to sustain motility in the thicker smooth muscle coat. Overall, this study expands our knowledge of the intrinsic innervation that regulates intestinal secretion, absorption and motility in gilthead sea bream and provides useful background information for rational design of functional feeds aimed at improving fish gut health.

## Introduction

The control of the main digestive functions in fish, as in other vertebrates, largely depends upon the activation of intrinsic neuronal circuitries constituting the enteric nervous system (ENS). The ENS is organized into two major plexuses, the myenteric and submucosal plexus. The myenteric plexus lays between the longitudinal and circular layers of the *muscularis propria* and is principally involved in the regulation of the gut motor function, whereas the submucosal plexus, which is less well developed in fish than in mammals, mainly regulates intestinal secretion [1,2]. The structural organization of fish myenteric plexus shows some peculiar differences with respect to the mammalian ENS. This latter consists of ganglia, composed of neurons and enteric glia, neuronal connections between ganglia, and nerve fibers supplying the effector tissues [3,4]. The fish ENS lacks such a well-organized network of ganglia and interconnecting fibers, instead, neurons are either scattered upon the longitudinal muscular layer or aggregated in small groups at the nodes of fiber connections. The pattern of neuron distribution over the muscular layer, however, is apparently not casual but follows nerve bundles along the length of the gut [1,2].

In mammals, activation of specific classes of neurons in the myenteric and submucosal plexuses underlies regulation of motor patterns, visceral sensory, secretory and absorptive functions, blood flow and interaction with the immune and enteroendocrine systems. The ENS modulates these digestive functions in a relative autonomous mode with respect to the central nervous system [5,6]. In the mammalian intestine there are about 20 neurochemically, functionally and morphologically identified types of enteric neurons, which constitute the three major classes of enteric neurons: motor neurons, intrinsic primary afferents neurons and interneurons [4,6,7]. Immunohistochemical approaches have been principally used to characterize the fish ENS, and there is now convincing data demonstrating that the principal neurotransmitter pathways found in the mammalian gut, are also present in the fish gut. These comprise both excitatory and inhibitory neurons innervating the longitudinal and circular smooth muscle. The main excitatory neurochemicals present in fish gut smooth muscle are acetylcholine and tachykinins (ACh, SP, and neurokinin A), whereas inhibitory pathways involve nitric oxide (NO) as the main transmitter and, to a minor extent, vasoactive intestinal peptide (VIP)-like and pituitary adenylate cyclase-activating polypeptide (PACAP)-like transmitters [8–11]. In the mammalian myenteric plexus, tachykinins, ACh and calcitonin gene-related peptide (CGRP) are recognized as the neurotransmitters in intrinsic sensory neuronal pathways, which participate in local reflexes by responding to chemical or mechanical stimuli within the gut lumen by sending inputs to the external musculature [4]. Serotonin (5-HT), released from enterochromaffin cells of the mucosa, was thought to be required for the activation of intrinsic primary afferent neurons in mammals. However, recent studies have shown that depletion of all endogenous 5-HT from the gut does not block peristalsis [12], nor reduce transit in vivo [13]. In fish myenteric plexus, however, both CGRP and 5-HT are mainly considered as neurochemicals modulating smooth muscle motor responses [14–16].

Despite previous findings, the chemical coding of fish ENS has not been systematically investigated as in other animals and only sparse data are available on the main neurotransmitter pathways present in the gut of some fish species, such as gilthead sea bream (*Sparus aurata*). This is one of the most farmed and economically relevant fish species for the Mediterranean aquaculture together with European sea bass (*Dicentrarchus labrax*) [17]. A detailed study of the gut neurophysiology in *S*. *aurata* may help to elucidate the mechanisms underlying intestinal digestion of nutrients, not only in wild fish, but also in fish reared in aquaculture conditions. Fish gut physiological functions are highly influenced by several factors, including diet and feeding habits. Such influence seems to be particularly important in teleost species due to different feeding habits (herbivorous, carnivorous or omnivorous), and different gastrointestinal structure (absence/presence of stomach, presence of pyloric ceca) [18]. Thus, the aim of this study was to evaluate the distribution of some of the most important enteric neurotransmitters in the myenteric plexus of gilthead sea bream. To this end, we immunohistochemically investigated the presence of nitrergic, serotoninergic, cholinergic, peptidergic (CGRP, SP, VIP) neurons in the proximal and distal intestine of juvenile fish. We studied the myenteric plexus chemical coding in these gut regions, given that the proximal and distal fish intestine have peculiar digestive functions [19] and may display different sensitivity to changes in the diet composition [20].

## Materials and methods

### Animals and tissue sampling

Gilthead sea bream (*Sparus aurata*) juveniles were reared in Nuova Azzurro hatchery (Civitavecchia, RM), in 2 m^3^ tanks, supplied with filtered sea water (37 g/l of salinity) at a temperature of 21.2 ± 1.4°C, and dissolved oxygen levels of 11.7 ± 0.6 mg/l. Three juvenile gilthead sea bream weighing 60.68 ± 0.84 g were used for the study. After 10 days of acclimatization during which fish were maintained under natural photoperiod and were fed to visual satiety with a balanced control diet for energy (17.5 MJ kg^-1^), protein (50%), and lipid (16%) content, fish were rapidly anaesthetized with tricaine methansulfonate (MS222, 300 ppm) and euthanized. The whole intestine was rapidly dissected out, and the proximal intestine was separated from the distal intestine. Intestinal segments were then rinsed with an ice-cold Tyrode’s solution (composition [mM]: 137 NaCl; 2.68 KCl; 1.8 CaCl_2__2H_2_O; 2MgCl_2_; 0.47 NaH_2_PO_4_; 11.9 NaHCO_3_; 5.6 glucose) and processed for histochemistry and immunofluorescence investigations. Animal care and handling were in accordance with the European Union Council Directive 2010/63, recognized and adopted by the Italian Government (DLgs No. 26 /2014). The protocol was approved by the Animal Care and Use Ethics Committee of the University of Insubria (n°03_2017).

### Histochemistry

The proximal and distal intestine of three gilthead sea breams were histologically evaluated by Hematoxylin and eosin (HE) histological staining. To this end, full-thickness intestinal samples were fixed with 4% formaldehyde for 24 h, dehydrated with a graded ethanol series (20, 30, 50, 70, and 95%), and then embedded in paraffin. HE histological staining was carried out on four-micron-thick sections and observed under a light microscope (Zeiss, West Germany). Data were recorded using a digital camera system (Discovery C30) and elaborated by a supporting software (ISCAPTURE). Cross sections of the proximal and distal intestine were used to evaluate the following morphological variables: thickness of the circular and longitudinal muscle and of submucosa layer, height and density of intestinal villi. The villus density was evaluated in seven transverse sections for each intestinal tract.

### Immunohistochemistry

Segments of the proximal and distal gilthead sea bream intestine were closed at one end with a nylon thread ligature, filled with 0.2 M sodium phosphate-buffer (PBS composition [M]: 0.14 NaCl, 0.003 KCl, 0.015 Na_2_HPO_4_, 0.0015 KH_2_PO_4_, pH 7.4) containing 4% formaldehyde plus 0.2% picric acid, and closed at the other end with a second nylon thread ligature. Preparations were then soaked in the same solution and fixed for 3 h at room temperature (RT). Following fixation, tissue preparations were cleared of fixative with 3 x 10-min washes in PBS and stored at 4°C in PBS containing 0.05% 2-(ethylmercuriomercapto) benzoic acid (thimerosal). Longitudinal muscle myenteric plexus (LMMP) whole-mount intestinal preparations were prepared according to the method of Giaroni et al. [21]. Briefly, small fixed intestinal segments (about 0.5 cm) were cut along the mesenteric border and pinned mucosal side upwards onto strips of Sylgard silicone rubber (Dow Corning, Seneffe, Belgium). After gently removing the mucosa with a scalpel, the submucosal and circular muscle layers were peeled away with fine forceps in order to obtain the longitudinal muscle with the myenteric plexus attached. LMMPs were then exposed to a PBS solution containing 1% Triton X-100 and 10% normal horse serum (NHS) for 1 h at RT (Euroclone, Celbio, Milan, Italy), to permeabilize the tissue and to block non-specific binding sites. Successively, tissues were incubated with optimally diluted primary antibodies (Table 1). Double labelling was performed during consecutive incubation times. Following overnight incubation at 4°C with the first primary antibody, preparations were washed 3 times, each for 5 min with PBS. LMMPs were then incubated for either 1 h at RT with an appropriate biotinylated secondary antibody followed by 1 h incubation with a streptavidine Cy3 or for 2 hours with a secondary antibody directly conjugated to a fluorophore (Table 1). A second primary antibody was then incubated overnight at 4°C and, after 3 x 5-min washes in PBS, the appropriate secondary antibody was added as aforementioned. Preparations were then given 3 x 10-min washes in PBS, before being mounted onto glass slides, using a commercially available mounting medium with DAPI (Vectashield^®^, Vector Lab., Burlingame, CA). All primary and secondary antibodies used in this study were commercially available. Specific features and working dilutions of both primary and secondary antibodies are shown in Table 1. Neuron counts were made on HuC/D stained LMMPs, obtained from three animals, digitized by capturing as many as 40 x objective microscope fields (0.1406 mm^2^) as possible (5–10 fields). The neuron count obtained for each field was divided by the total image field area and expressed as the number of neurons/mm^2^ (a total of 45 and 60 fields were counted for the proximal and distal intestinal region, respectively). Neuronal cell body area was measured with Image J NIH image software (http://imagej.nih.gov/ij) [22]. The distribution of soma sizes of HuC/D stained myenteric neurons (n = 250 total from three fish) has been evaluated by counting how many values fell into each consecutive interval of 20 μm^2^. The size of neuronal cell bodies were arbitrarily divided into small (with an area less than 100 μm^2^), medium (with an area ranging between 100–250 μm^2^) and large (with an area ranging between 260–600 μm^2^).

**Table 1:** Primary and secondary antisera used and respective dilutions.

Antiserum	Dilution	Source	Host species
**Primary antisera**			
HuC/D Biotin	1:100	Invitrogen (16A11)	Mouse
VIP	1: 200	Immunostar (20077)	Rabbit
5-HT	1:200	Immunostar (20079)	Goat
CGRP	1:200	Immunostar (24112)	Rabbit
Substance P	1:200	Immunostar (20064)	Rabbit
Calbindin (D-28k)	1:500	Swant (CB-38a)	Rabbit
ChAT	1:150	Abcam (ab70219)	Rabbit
nNOS (R-20)	1:50	Santa Cruz (sc-648)	Rabbit
**Secondary antisera & streptavidin complexes**			
Anti-mouse Alexa Fluor 488	1:150	Molecular Probes (A21202)	Donkey
Anti-rabbit Alexa Fluor 488	1:250	Molecular Probes (A21206)	Donkey
Cy3-conjugated streptavidin	1:500	Amersham (PA43001)	
Anti-goat Cy3-conjugated	1:500	Jackson ImmunoResearch (705-165-147)	Donkey

**Supplying companies:** Abcam, Cambridge, UK; Amersham, GE Healthcare, Buckinghamshire, UK; Jackson Immuno Research Laboratories, Inc., Baltimore, USA; Molecular Probes, Invitrogen, Carlsbad, CA, USA; Santa Cruz Biotechnology, Inc., CA, USA. Invitrogen, Thermo Fisher Scientific.

To establish the proportion of nNOS, 5-HT, ChAT, SP, CGRP and VIP expressing myenteric neurons, quantitative analysis of double fluorescently labelled small intestine whole mounts was performed as previously described [23]. Briefly, the number of neurons that was immunoreactive for the pan-neuronal marker, HuC/D was first counted and then the number of neurons that were immunopositive to the second antibody labelled with a fluorophore of a different colour was determined. A total of 5–15 fields was sampled from LMMP preparations obtained from the proximal and distal intestine of three animals. Some experiments were carried out to evaluate the possible co-localization of 5HT and CGRP with the Ca^++^-binding protein calbindin. Calbindin, is considered as a marker for sensory neurons in the mammalian ENS, and is highly co-expressed in 5-HT containing myenteric neurons in the fish gut [4, 24, 25]. Histograms showing the distribution of the soma sizes of nNOS, ChAT, 5-HT, SP, CGRP and VIP-expressing neurons were constructed by counting how many values fell into each consecutive interval of 20 μm^2^. Negative controls and interference control staining were evaluated by omitting both primary and secondary antibody, and by incubating colonic whole-mounts with non-immune serum from the same species in which the primary antibodies were raised. In all these conditions, no specific signal was detected. Preparations were analysed using a Leica TCS SP5 confocal laser scanning system (Leica Microsystems GmbH, Wetzlar, Germany) and pictures were processed with Adobe-Photoshop CS6S software.

### Statistics

All data are expressed as mean ± standard error of the mean (SEM) with 95% confidence interval (CI),except for the data on neuronal area, expressed as mean area ± standard deviation (SD) with 95% CI A randomized blocks ANOVA analysis was performed to evaluate differences among the number of HuC/D positive neurons in the three fish. Percentage variations expressing the proportions of different myenteric neuron populations in the proximal and distal intestine have been compared after angular transformation. Statistical significance was calculated with Student’s t test for unpaired data using GraphPad Prism (version 5.3 GraphPad software, San Diego, CA, USA). Differences between groups were considered significant when *P* value is 0.05 or lower. Statistical significance was expressed with the following symbols: (*), (**) and (***), to indicate that differences between groups were statistically significant, highly significant or extremely significant, respectively.

### Materials

All chemicals were purchased from Sigma Aldrich (Milan, Italy), except for primary and secondary antibodies (see Table 1).

## Results

**Fig 1:**
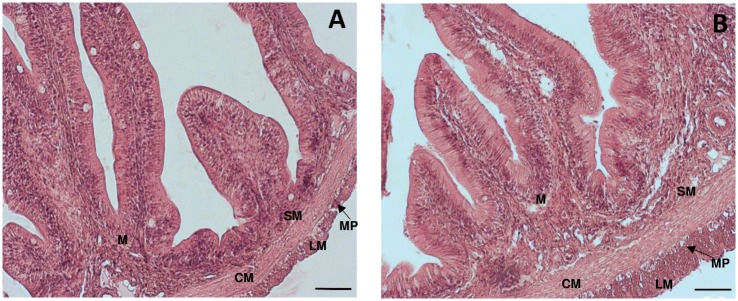
Hematoxylin-eosin (HE) staining in cross sections of the proximal (A) and distal (B) guilthead sea bream intestine. **M**, mucosa; **SM**, submucosa; **CM,** circular muscle layer; **LM** longitudinal muscle layer; **MP**, myenteric plexus. (Bar 50 μm).

Intestinal cross-sections revealed morphological differences in the structure of the epithelium of the two target regions. In particular, in the proximal intestine, villi were generally branched and characterized by an elongated shape projecting into the intestinal lumen, whereas in the distal intestine villi were stubbier with a larger base (Fig 1, panels A-B). The number of villi per cross-section was significantly higher in the proximal intestine with respect to the distal intestine (Table 2). The submucosal layer was significantly thicker in the proximal than in distal intestine (Table 2). The submucosal plexus was not evident within this layer. The thickness of internal circular smooth muscle was similar in the two regions studied, whereas the longitudinal smooth muscle layer was significantly thicker in the distal than in the proximal intestine (Table 2). The total smooth muscle layer thickness of the distal intestine was higher than in the proximal intestine (Table 2). In both regions, the myenteric plexus was evident between the circular and longitudinal smooth muscle layer (Fig 1, panels A-B).

**Table 2:** Intestinal morphology parameters of the proximal and distal gilthead sea bream intestine.

	PROXIMAL INTESTINE	DISTAL INTESTINE
Smooth muscle thickness (μm)	45.50 ± 2.72(39.87–51.13) (n = 24)	55.60 ± 4.08 (47.14–64.03) (n = 25) *
Circular muscle thickness (μm)	26.10± 2.42 (21.05–31.14) (n = 21)	29.50 ± 1.80 (25.80–33.20) (n = 26)
Longitudinal muscle thickness (μm)	18.24 ± 1.16 (15.82–20.66) (n = 21)	22.40 ±2.32 (21.05–31.14) (n = 21) **
Submucosa thickness (μm)	17.81 ± 1.30 (15.04–20.59) (n = 16)	11.44 ± 0.84 (9.69–13.18) (n = 16) ***
Villi height (μm)	345.48 ± 27.58 (288.8–402.2) (n = 27)	341.67± 17.38 (305.9–377.4) (n = 27)
Villi density	45.43 ± 3.14 (37.73–53.13) (n = 7)	31.43 ±2.39 (25.56–37.30)** (n = 7)

Values are reported as means of ± SEM with 95% CI (n indicates the total number of measurements obtained in the proximal and distal intestine of 3 fish. Villi density was calculated as number of villi per transverse section (n = 7) for each intestinal region.

***P≤0.001,

**P≤0.01,

*P≤0.05 vs values obtained in the proximal intestine by Student’s t test.

### General description of myenteric neurons in the proximal and distal gilthead sea bream intestine

**Fig 2:**
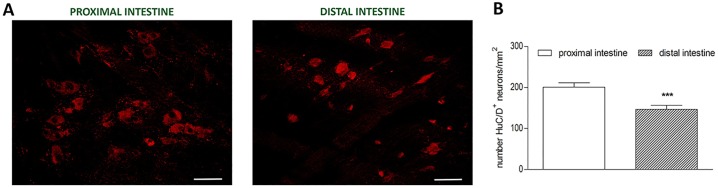
Density of myenteric neurons in longitudinal muscle myenteric plexus (LMMP) whole mounts of the proximal and distal gilthead sea bream. (**A**) HuC/D staining in LMMP preparations of the proximal and distal gilthead sea bream intestine. Bars: 50 μm. (**B**) Number of myenteric neurons per mm^2^ staining for HuC/D in gilthead sea bream proximal and distal intestine. Values are given as mean ± SEM (n = 45 and n = 60, respectively). ***P≤ 0.001 vs proximal intestine by Student’s t test.

**Fig 3:**
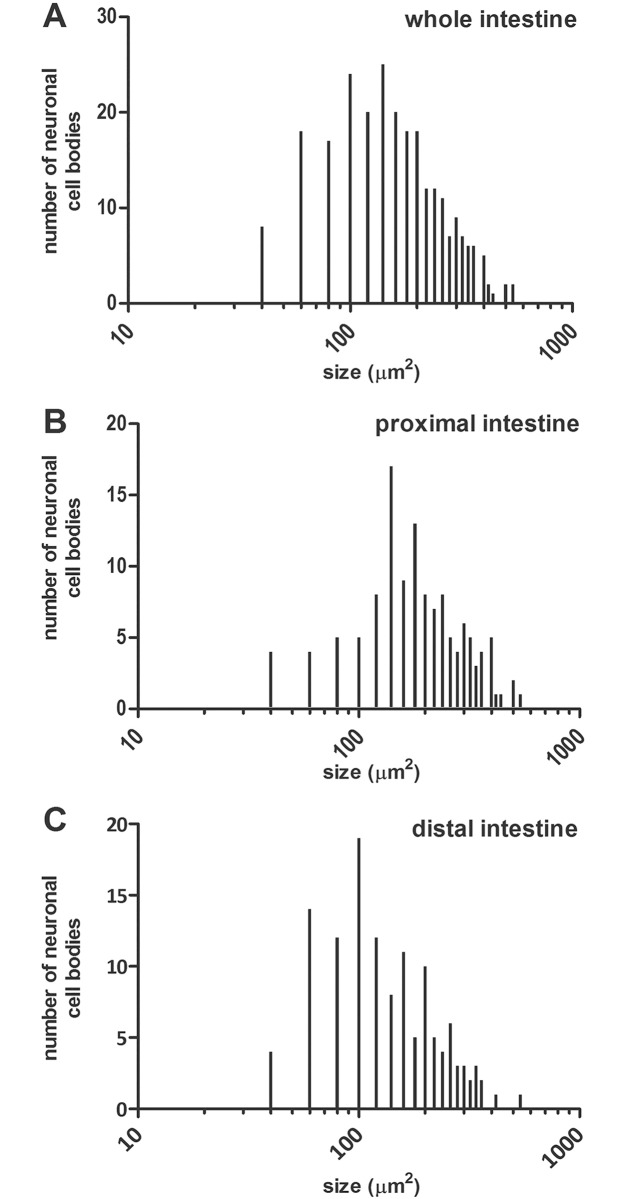
Size distribution of neuronal cell bodies in the gilthead sea bream intestine as determined after HuC/D staining in LMMP whole mounts preparations. (**A**) Myenteric neuron cell body size distribution in the whole intestine. Panels **B** and **C** show the size distribution in the proximal and distal intestine, respectively.

In LMMP preparations of the proximal and distal gilthead sea bream intestine the neuronal marker HuC/D stained the soma of all myenteric neurons. In both intestinal regions, myenteric neurons appeared as single neuronal cells distributed over the longitudinal layer, although, small aggregates, consisting of 3–8 cells were also detectable (Fig 2, panel A). The number of myenteric neurons, normalized per area (mm^2^), was significantly lower (*P*≤0.001) in the distal intestine [146.8±9.67 (127.4–166.1), n = 45] with respect to the proximal intestine [200.9±10.46 (179.8–222.0), n = 60] (Fig 2 panel B). No significant differences were obtained for the number of HuC/D labelled neurons among fishes after randomized blocks ANOVA (data not shown). In both proximal and distal intestine, the size of myenteric neuron cell bodies varied reflecting the presence of different neuronal populations. In the whole intestine, neuronal cell body area ranged from a minimum mean value of 71.7 ± 16.55 (66.82–76.54) μm^2^ (n = 47 from three fish) to a maximum mean value of 335.6 ± 71.29 (317.1–354.2) μm^2^ (n = 59 from three fish). In the proximal intestine, the average cell body size was 213.08 ± 105.46 (195.1–232.5) μm^2^ (n = 125 cells from three fish), whereas in the distal intestine the mean area of cell bodies was significantly lower [162.63 ± 90.98 (146.5–178.7) μm^2^, P≤0.001, n = 125 cells from three fish] with respect to the value obtained in the proximal intestine (Table 3). In particular, the number of neurons with a small soma size, conventionally established as lower than 100 μm^2^, in the distal intestine was more than double the number obtained in the small intestine. In addition, in the distal intestine, the mean area of the medium sized neurons (ranging from 100 to 250 μm^2^) was significantly lower than in the proximal intestine (Fig 3 and Table 3).

**Table 3:** Average size of myenteric neurons in the proximal and distal gilthead sea bream intestine.

SIZE	PROXIMAL INTESTINE	DISTAL INTESTINE
Small (<100 μm^2^)	64.80 ± 18.23 (54.31–75.40) (n = 12)	74.3 ± 15.68 (68.82–79.76) (n = 34)
Medium (100–250 μm^2^)	173.6 ± 45.58 (162.9–183.70) (n = 76)	156.3 ± 40.61 (146.4–165.9) * (n = 69)
Large (260–600 μm^2^)	345.2 ± 72.54 (321.0–369.4 (n = 37)	319.5 ± 67.69 (289.5–349.5) (n = 22)
Average size	213.08 ± 105.50 (195.1–232.5) (n = 125)	162.63 ± 90.97 (146.5–178.7)*** (n = 125)

Values are expressed as mean area ± SD with 95% CI, with n indicating the number of neurons.

***P≤0.001,

*P≤0.05 vs proximal by Student’s t test.

### Distribution of nNOS immunoreactivity in the proximal and distal gilthead sea bream intestine

**Fig 4:**
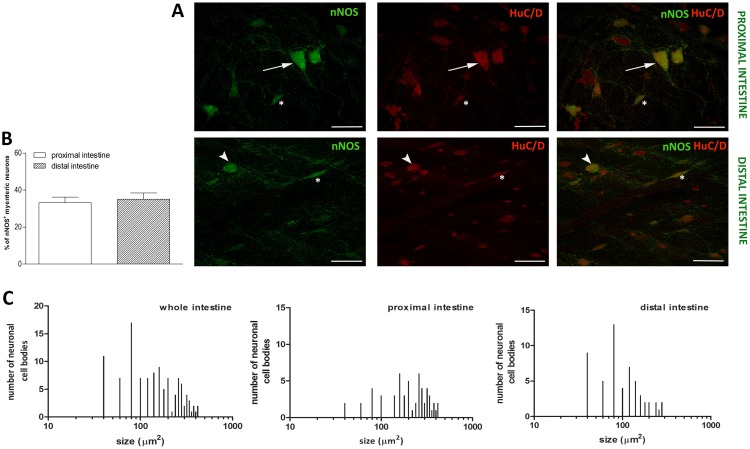
Distribution of nNOS in LMMP whole mounts of the proximal and distal gilthead sea bream intestine. (**A**) Immunohistochemical co-localization of nNOS with HuC/D. In both regions, nNOS-IR neurons displayed a variable size and morphology. Some neurons displayed a large polygonal soma, one long axon and short dendrites (arrow). Other nNOS-IR neurons were unipolar with a rounded soma and short dendrites (arrowhead). Few neurons displayed an elongated oval soma (asterisk). nNOS-IR was also found in neuronal fibers running along the smooth muscle layer. Bars: 50 μm. (**B**) Percentage of nNOS immunoreactive neurons in the proximal (empty bar) and distal (slashed bar) gilthead sea bream intestine. Values are given as mean ± SEM of 15 fields for each intestinal region. (C) Size distribution of the cell body of nNOS immunoreactive neurons in the whole, proximal and distal gilthead sea bream intestine.

Nitrergic myenteric neurons in the gilthead sea bream intestine have been immunohistochemically characterized using a specific antibody recognizing neuronal nitric oxide (NO) synthase (nNOS). In LMMP whole-mount preparations of both proximal and distal intestine, nNOS immunoreactivity (nNOS-IR) was observed in the soma of either single neurons or neurons forming groups of two-three cells. The intensity of staining was very high in some neurons and moderate in others. nNOS-IR was also evidenced in few nerve fibers running along the longitudinal smooth muscle layer (Fig 4, panel A).

The percentage of nNOS positive myenteric neurons, determined by co-staining with HuC/D, in the proximal intestine [33.26± 2.93 (26.98–39.55) %, n = 15 fields] was not significantly different with respect to the value obtained in the distal intestine [35.06± 3.36 (27.43–42.65) %, n = 15 fields] (Fig 4 panel B). In the whole gilthead sea bream intestine, the size of nNOS-IR neurons varied from small [68.8±18.63 (62.77–74.85) μm^2^, n = 39] to large neurons [334.6± 96.77 (297.1–372.2) μm^2^_,_ n = 28] (Fig 4, panel C). Some neurons displayed a medium or large polygonal body with short flattened dendrites and one long axon. Other large uniaxonal neurons had a rounded soma with short dendrites. In both regions, some neurons of small and medium size displayed an elongated oval shape with no visible dendrites (Fig 4, panel A). nNOS-IR neurons with larger size were mainly located in the proximal intestine, whereas the distal region displayed a higher number of small size neurons (Fig 4, panel C). The average size of neuron cell bodies in the proximal intestine was significantly higher with respect to the value obtained in the distal intestine [232.24 ± 127.1 (198.2–266.3) μm^2^, n = 56; 113.5 ± 63.4 (96.31–130.6) μm^2^, n = 55, respectively, *P*<0.001].

### Distribution of VIP immunoreactivity in the proximal and distal gilthead sea bream intestine

**Fig 5:**
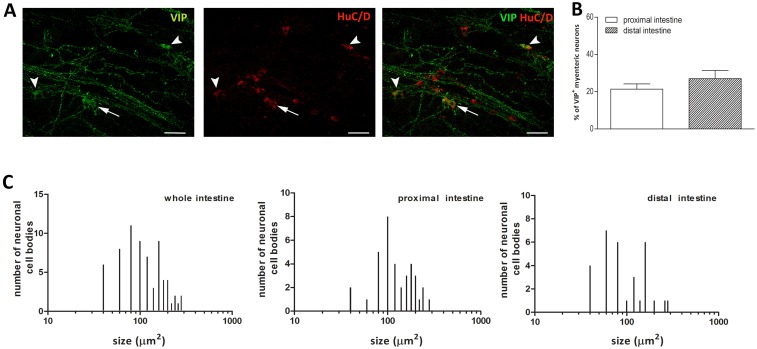
Distribution of VIP in LMMP whole mounts of the proximal and distal gilthead sea bream intestine. (**A**) Immunohistochemical co-localization of VIP with HuC/D. VIP immunoreactivity was detected in few neuronal cell bodies with either a round soma and short dendrites (arrow) or a smooth oval soma (arrowhead). Bars: 50 μm. (**B**) Percentage of VIP-IR neurons in the proximal (empty bar) and distal (slashed bar) gilthead sea bream intestine. Values are given as mean ± SEM of 5–6 fields for each intestinal region. Vertical bars indicate SEM. (**C**) Size distribution of the cell body of VIP-IR neurons in the whole, proximal and distal intestine of gilthead sea bream intestine.

VIP immunoreactivity was predominantly seen in bundles of fibers along the longitudinal smooth muscle layer and in few myenteric neurons scattered along the longitudinal muscle (Fig 5, panel A). The percentage of VIP positive myenteric neurons, as determined by co-staining with HuC/D, was similar in the proximal and distal intestine [21.38± 2.78 (14.22–28.55)%, n = 6 fields; 27.03± 4.30 (15.08–38.97)%, n = 5 fields, respectively] (Fig 5, panel B). In the whole intestine, the size of VIP-IR neurons varied from small neurons [78.51± 17.24 (65.25–91.78) μm^2^, n = 9] to large neurons [278.2± 12.49(165.7–390.6) μm^2^, n = 2], however, most VIP-IR neurons were of medium size [157.50±41.51 (144.1–171.0) μm^2^, n = 39] (Fig 5, panel C). VIP-IR neurons had an oval soma with no visible dendrites or a rounded soma with visible dendrites. Intense VIP immunoreactivity was also found fibers innervating the longitudinal muscle. VIP-IR fibers were varicose and surrounded both VIP-positive and VIP-negative neurons. The average size of neuronal cell bodies in the proximal intestine was not significantly different with respect to the value obtained in the distal intestine [140.10 ± 55.99 (121.2–159) μm^2^, n = 36; 115.60 ± 59.66 (93.74–137.5) μm^2^, n = 31] (Fig 5, panel C).

### Distribution of 5-HT immunoreactivity in the proximal and distal gilthead sea bream intestine

**Fig 6:**
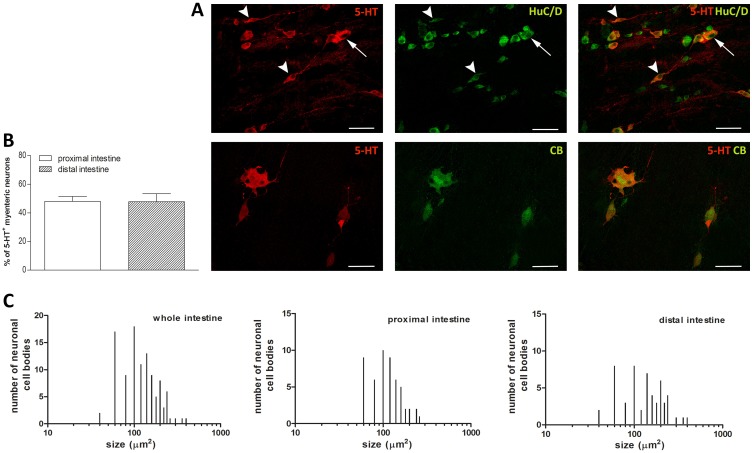
Distribution of 5-HT in LMMP whole mounts of the proximal and distal gilthead sea bream intestine. (**A**) Immunohistochemical co-localization of 5-HT with the neuronal marker HuC/D and with calbindin (CB). 5-HT immunoreactivity was detected in the soma and axon of myenteric neurons with a large rounded soma and visible dendrites (arrow). Some 5-HT-IR neurons displayed an elongated soma (arrowheads) with a long axon. The co-localization between 5-HT and CB was almost total. Staining Bars: 50 μm. (**B**) Percentage of 5-HT-IR neurons in the proximal (empty bar) and distal (slashed bar) sea bream intestine. Values are given as mean ± SEM of 7–11 fields for each intestinal region. Vertical bars indicate SEM. (**C**) Size distribution of the cell body of 5-HT-IR neurons in the whole, proximal and distal intestine gilthead sea bream intestine.

5-HT immunoreactivity was predominantly found in the soma of either single neurons or neurons forming groups of two-four cells (Fig 6, panel A). The percentage of 5-HT positive myenteric neurons as determined by co-staining with HuC/D was similar in the proximal and distal intestine [48.07± 3.43 (39.95–56.19) %, n = 8 fields and 47.83±5.64 (34.03–61.63) %, n = 7 fields, respectively] (Fig 6, panel B). In the whole intestine, the size of 5-HT-IR neurons varied from small [73.1±17.44 (67.50–78.8) μm^2^, n = 39] to large neurons [329.7 ± 61.18 (232.3–427.1) μm^2^, n = 4] (Fig 6, panel C), the majority of 5HT-IR reactive neurons measured were of medium size [155.8 ± 44.38 (144.5–167.1) μm^2^, n = 62]. Some neurons had a large rounded body with short dendrites and a long axon. Other serotoninergic neurons displayed an oval soma with no visible dendrites and with a long varicose and non-varicose axon (Fig 6, panel A). The single processes arising from a 5-HT nerve cell body were of variable length, some being very long and traceable for a long distance from the cell of origin. In general, such processes run along the length of the longitudinal muscle without terminating around both 5-HT-IR or non-immunoreactive neural cell bodies. The average size of neuronal cell bodies in the proximal intestine was significantly higher with respect to the value obtained in the distal intestine [145 ± 10.80 (123.3–166.6) μm^2^, n = 53; 118.2 ± 50.27 (104.2–132.2) μm^2^, n = 52, respectively, P≤0.05) (Fig 6, panel C). The majority of 5-HT immunopositive myenteric neurons both in the proximal and distal intestine was also immunopositive to the Ca^++^ binding protein, calbindin [91.22±3.66 (83.06–99.38) %, n = 11 fields; 88.79±3.34 (81.25–96.34)%, n = 10 fields, respectively] (Fig 6 panel A).

### Distribution of CGRP immunoreactivity in the proximal and distal gilthead sea bream intestine

**Fig 7:**
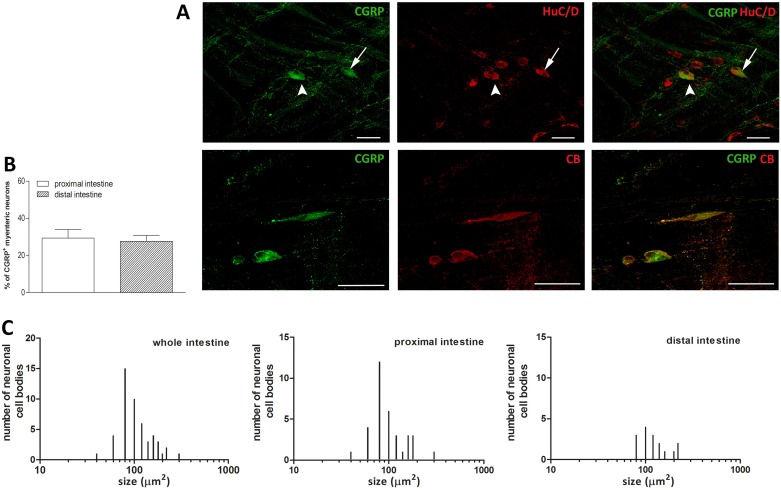
Distribution of CGRP in LMMP whole mounts of the proximal and distal gilthead sea bream intestine. (**A**) Immunohistochemical co-localization of CGRP with the neuronal marker HuC/D and with calbindin (CB). In both proximal and distal region, CGRP immunoreactivity was present in thinner and thicker bundles of fibers running along the longitudinal smooth muscle layer. CGRP immunoreactivity was also detected in medium size neurons displaying a smooth soma with no visible dendrites and an oval shape (arrow), some neurons were multipolar (arrowhead). Bars: 50 μm. (**B**) Percentage of CGRP immunoreactive neurons in the proximal (empty bar) and distal (slashed bar) gilthead sea bream intestine. Values are given as mean ± SEM of 7–10 fields for each intestinal region. Vertical bars indicate SEM. (**C**) Size distribution of the cell body of CGRP neurons in the whole, proximal and distal gilthead sea bream intestine.

CGRP immunoreactivity was predominantly seen in fibers and in neurons scattered along the longitudinal smooth muscle layer (Fig 7, panels A). The percentage of CGRP positive myenteric neurons as determined by co-staining with HuC/D was similar in the proximal and distal intestine [29.39± 4.59 (18.15–40.63) %, n = 7 fields and 27.72±3.15 (20.02–35.43) %, n = 7 fields, respectively] (Fig 7, panel B). In the whole intestine, the size of CGRP-IR neurons varied from small neurons (78.05± 12.35 (72.43–83.67) μm^2^, n = 21] to large neurons [310.8± 13.90 (276.2–345.3) μm^2^, n = 3], however, most CGRP-IR neurons were of medium size [133.4±36.85 (118.5–148.3) μm^2^, n = 32] (Fig 7, panel C). In both intestinal regions, CGRP-IR neurons had predominantly an oval soma with no visible dendrites, some neurons displayed multiple axons. CGRP immunoreactivity was also found in varicose fibers innervating the longitudinal muscle (Fig 7, panel A). The average size of neuronal cell bodies in the proximal intestine was not significantly different with respect to the value obtained in the distal intestine [108.40 ± 49.58 (91.08–125.7) μm^2^, n = 34; 132.70 ± 75.81 (103.3–162.1) μm^2^, n = 28, respectively] (Fig 7, panel C). The majority of CGRP immunopositive myenteric neurons both in the proximal and distal intestine was also immunopositive to the Ca^++^ binding protein, calbindin [90.12±3.83 (81.45–98.79) %, n = 10 fields; 91.60±3.18 (84.41–98.79)%, n = 10 fields, respectively) (Fig 7, panel A).

### Distribution of ChAT immunoreactivity in the proximal and distal gilthead sea bream intestine

**Fig 8:**
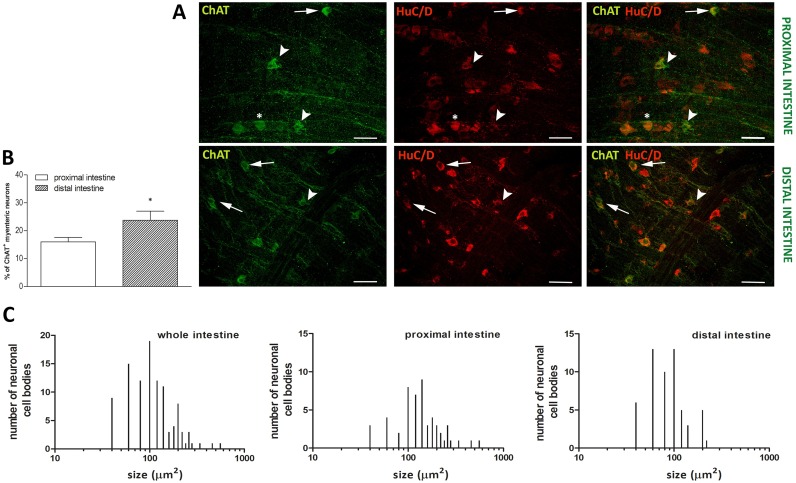
Distribution of ChAT in LMMP whole mounts of the proximal and distal gilthead sea bream intestine. (**A**) Immunohistochemical co-localization of ChAT with the neuronal marker HuC/D. Both in the proximal and distal intestine, ChAT immunoreactivity was found in myenteric neurons and in fibers oriented in longitudinal direction. Some ChAT-IR uniaxonal neurons displayed a smaller-medium range size oval soma with smooth contours (arrow). Other neurons in the medium range size had an irregular profile with broad lamellar dendrites (arrowhead), and a small number of neurons had smooth, round or oval cell bodies (asterisk). Bars: 50 μm. (**B**) Percentage of ChAT-IR neurons in the proximal (empty bar) and distal (slashed bar) gilthead sea bream intestine. Values are given as mean ± SEM of 9–14 fields sections for the proximal and distal region, respectively. Vertical bars indicate SEM, *P≤0.05 by Student’s t test. (**C**) Size distribution of the cell body of ChAT-IR neurons in the whole, proximal and distal gilthead sea bream intestine.

Cholinergic myenteric neurons in the gilthead sea bream intestine have been immunohistochemically characterized using an antibody specifically recognizing the acetylcholine-synthesizing enzyme, choline acetyl transferase (ChAT). ChAT antiserum stained the soma of sparse myenteric neurons and fibers running along the longitudinal smooth muscle layer (Fig 8, panel A). In the distal intestine, the percentage of ChAT positive myenteric neurons was significantly higher with respect to the value obtained in the proximal intestine [proximal: 16.00±1.66 (12.42–19.58)%, n = 14 fields; distal: 23.74±3.89 (14.77–32.72)%, n = 9 fields, respectively *P*≤0.05] (Fig 8, panel B). In the whole intestine, the size of ChAT-IR neurons varied from small neurons [67.9± 19.12 (62.16–73.65) μm^2^, n = 45] to large neurons [346.2± 115.29 (239.6–452.8) μm^2^, n = 7], most of ChAT reactive neurons measured were of medium size [150.10 ± 39.77 (139.5–160.6) μm^2^, n = 57] (Fig 8, panel C). Myenteric neurons staining for ChAT displayed a variety of shapes. Neurons in the smaller-medium range were usually oval smooth and uniaxonal neurons. Another neuronal population in the medium range had an irregular profile with broad lamellar dendrites, while a small number of neurons had a smooth, round or oval cell bodies that showed a pale staining (Fig 8, panel A). In the proximal intestine, the average size of ChAT-IR myenteric neurons was significantly higher than in the distal intestine [161 ± 94.86 (135.3–187.6) μm^2^, n = 53; 97.82 ± 48.61 (84.80–110.8) μm^2^, n = 56, respectively, *P*≤0.001) (Fig 8, panel C).

### Distribution of SP immunoreactivity in the proximal and distal gilthead sea bream intestine

**Fig 9:**
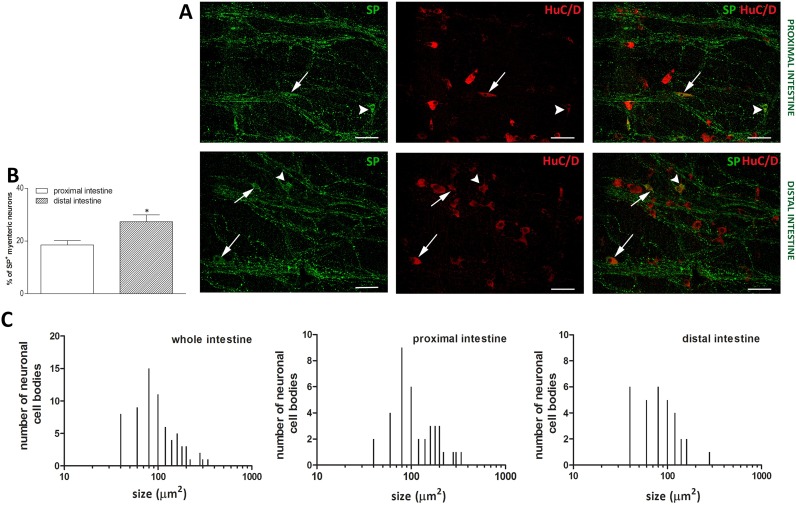
Distribution of SP in LMMP whole mounts of the proximal and distal gilthead sea bream intestine. Immunohistochemical co-localization of SP with the neuronal marker HuC/D in whole mounts of the proximal and distal gilthead sea bream intestine. (**A**) Both in the proximal and distal intestine, SP immunoreactivity was detected in fibers running along the smooth muscle layers (asterisks) and in the soma of sparse neurons displaying either an elongated (arrow) or a rounded soma with short dendrites (arrowhead). Bars: 50 μm. (**B**) Percentage of SP immunoreactive neurons in the proximal (empty bar) and distal (slashed bar) gilthead sea bream intestine. Values are given as mean ± SEM of 7 fieldsfor each intestinal region. Vertical bars indicate SEM; *P≤0.05 by Student’s t. (**C**) Size distribution of the cell body of SP neurons in the whole, proximal and distal intestine gilthead sea bream intestine.

SP-IR was generally faint in the soma of scattered myenteric neurons and more intense in trunks of varicose fibers along the longitudinal muscle (Fig 9, panel A). The percentage of myenteric neurons staining for SP was significantly higher (*P*≤0.05) in the distal than in the proximal intestine [proximal: 18.56 ± 1.61 (14.61–22.51)%, n = 7 fields; distal: 27.34± 2.62 (20.92–33.75) %, n = 7 fields] (Fig 9, panel B). In the whole intestine, the majority of SP-IR neurons had either a small [69.3± 18.82 (63.03–75.59) μm^2^, n = 37] or medium size [146.80 ± 35.27 (133.2–160.5) μm^2^, n = 28] and few neurons were of large size [294.7± 34.11 (240.7–349) μm^2^, n = 4] (Fig 9, panel C). Some cells were uniaxonal and displayed an elongated soma with no visible dendrites. Another set of uniaxonal neurons displayed a rounded soma with short dendrites. The average size of neuronal cell bodies in the proximal intestine was significantly higher with respect to the value obtained in the distal intestine [132.9 ± 71.31 (109.1–156.7) μm^2^, n = 37; 92.20 ± 49.20 (74.15–110.2) μm^2^, n = 31, respectively, *P*≤0.01] (Fig 9, panel C).

## Discussion

In this study, we have identified for the first time the major neurochemicals present in enteric neurons of the intestine of the gilthead sea bream. The distribution of different neuronal populations in the two regions are largely superimposable, although some differences were observed, which might depend upon the morphological peculiarities of each region. Both in the proximal and distal gilthead sea bream intestine, myenteric neurons were distributed over the longitudinal muscle layer, either as single cells or as small aggregates of cells, as observed after staining with HuC/D, the pan neuronal marker for enteric neurons [26]. Neuronal density in the proximal and distal intestine was 200.90 cells/mm^2^ and 146.80 cells/mm^2^, respectively, being similar to the neuronal density observed in the myenteric plexus of two other teleosts fish, the brown trout (110–180 cells/mm^2^) and the Atlantic cod (*Gadus morhua*) (62–106 cells/mm^2^) [1, 27]. These data are in agreement with the density of myenteric neurons in the intestine of small mammals, such as the mouse [28]. The average size of neuronal cell profiles in the myenteric plexus of gilthead sea bream is also comparable to values obtained in the myenteric plexus of other teleosts [27] and in small mammals, such as rat, mouse and guinea pig [28,29]. Interestingly, in the distal intestine, both the neuronal density and the average neuronal size were significantly lower than in the proximal region. Accordingly, in the rat, the number of colonic myenteric neurons/mm^2^ was higher in the small intestine than in the colon [29,30]. In addition, Gomes et al. and Castelucci et al. [31, 32] demonstrated that the myenteric neuron profile area was greater in the small intestine than in the large intestine of rats. Variations in the density and size of enteric neurons have been related to the volume of the tissue innervated [28]. However, in *S*. *aurata*, the smooth muscle coat of the proximal intestine, which represents the main target of myenteric motor neurons, is less thick than in the distal intestine. This discrepancy may be explained considering that the myenteric plexus comprises several classes of neurons including primary afferents, secretomotor neurons and interneurons, which project to the mucosa and submucosal layer. Thus, the higher number and greater size of myenteric neurons in the gilthead sea bream proximal intestine may be related to the higher density and thickness of the submucosal and mucosal layers observed in this region, with respect to the distal intestine. Innervation of both submucosal and mucosal layers by myenteric neurons may be particularly important in fish, since the submucosal plexus is less well developed with respect to mammals.

The major neurochemicals previously reported to be present in the myenteric plexus of the intestine of fish and vertebrates are indeed present in the intestine of gilthead sea bream.

### Nitric oxide

Our immunohistochemical data show that approximately 33–35% of myenteric neurons both in the proximal and distal intestine express neuronal nitric oxide synthase (nNOS). nNOS represents the predominant source of NO produced by enteric neurons in physiological conditions [33] and is expressed in the intestine of mammals and in other vertebrate classes, including fish [34–36]. In mammals, the percentage of myenteric neurons producing NO is relatively stable, ranging from the 10% to the 25%, according to the species [37]. In contrast, in fish, the number of myenteric nitrergic neurons is more variable, resulting the 65% of the total neuronal population in the elasmobranch spiny dogfish, the 48% and 15–20% in the Atlantic cod (*Gadus morhua*) and rainbow trout (*Onchorhynchus mykiss*), respectively [10,27]. Such variability may depend upon the fish species, but also on the method used to label nitrergic neurons, which was formerly carried out using NADPH-diaphorase labeling, since NOS is a NADPH-diaphorase [38,39]. In this study, we used a specific antibody raised against nNOS and the percentage of nitrergic myenteric neurons wasin line with values obtained in the same fish class [10, 27]. In *S*. *aurata* intestine, few nerve fibers were nNOS immunoreactive, suggesting that nitrergic neurons may impinge on smooth muscle to modulate its function. These may represent anally projecting nNOS-reactive fibers involved in the descending inhibition of the peristaltic reflex [5, 40, 41]. The existence of a nitrergic inhibitory pathway in our model is strengthened by the presence of large uniaxonal neurons with short dendrites, described as putative inhibitory motor neurons to the smooth muscle. In accordance with data obtained in small mammals s, such as the rat, the density of nNOS-IR neurons with larger soma size was higher in the proximal intestine with respect to the large intestine [42,43]. The action of NO in the gut is however not limited to direct inhibition of the motor function but may also involve modulation of descending interneuronal pathways as well as the sensory function. In this view, as observed in the Atlantic cod intestine, the high morphological variability of nNOS immunoreactive cells in the gilthead sea bream intestine may depend on the multiple roles of nitrergic neurons in the modulation of the gut function [27].

### Vasoactive intestinal peptide

In the ENS of several mammals, vasoactive intestinal peptide (VIP), acts as an inhibitory neurotransmitter together with NO in the descending reflex of peristalsis [5,44]. In our study, the percentage of VIP-IR myenteric neurons was similar in the proximal and distal gilthead sea bream intestine, reaching approximately a percentage of 21–27%. These data agree with the number of VIP-IR neurons found in the myenteric plexus of mammals and fish [27,45]. VIP labelling in myenteric neurons was either weak or moderate, whereas nerve fibers displayed a strong staining along the direction of the longitudinal muscle. Similar results have been obtained in the Atlantic cod [27,41] and along the whole gastrointestinal tract of rainbow trout (*O*. *mykiss*) [46,47]. In the myenteric plexus of mammals, VIP is present in inhibitory motor neurons as well as in interneurons involved in the descending inhibitory reflex [45,48]. In the Atlantic cod intestine, the presence of anally projecting VIP-IR nerve fibers was demonstrated after myotomy [41]. In this study, the abundance of VIP-IR fibers surrounding both VIPergic and non-VIPergic neurons is suggestive of the presence of putative VIP containing descending interneurons also in the gilthead sea bream intestine. From a functional viewpoint however, there are no consistent data on the ability of VIP to directly modulate the fish intestinal motor function. In this view, the peptide is prevalently considered as a neuromodulator of inhibitory motor neurons more than as a neurotransmitter [11].

### Serotonin

Serotonin (5-HT) has long been considered to play a key role in the modulation of several gut functions and the intestinal sources of 5-HT are mainly represented by enterochromaffin (EC) cells of the mucosa and by myenteric neurons that project in the descending pathway of the peristaltic reflex [49]. Although it is important to note that depletion of all endogenous 5-HT does not block peristalsis in the large intestine of vertebrates [12]. In the mammalian intestine the main source for 5-HT is represented by EC cells, whereas in fish intestine most of 5-HT is stored in enteric nerves [49–51]. In our study, serotoninergic neurons represented the largest neuronal population, reaching an average percentage of about 48% both in the proximal and distal gilthead sea bream intestine. The density of 5-HT-IR myenteric neurons is particularly high also in other teleosts such as rainbow trout (*Oncorhynchus mykiss*), sand flathead (*Platycephalus bassensis*), smooth toadfish (*Tetractenos glaber*) and short-finned eel (*Anguilla australis*) [51,52]. The abundance of serotoninergic neurons in our study was paralleled by a high variability of the size and shape of 5-HT-IR myenteric neurons. Uniaxonal neurons with a large soma and short dendrites may represent myenteric motor neurons, consistent with previous findings obtained in the rat and guinea pig myenteric plexus [53,54]. In fish, 5-HT has been proposed to be an excitatory non-cholinergic transmitter to smooth muscle [55–57]. In addition, 5-HT may modulate fish intestinal smooth muscle activity by activating both ascending and descending interneuron pathways [58]. In our study, uniaxonal ovoidal 5HT-labelled neurons may represent a population of myenteric interneurons. In analogy with data obtained in the shorthorn sculpin myenteric plexus, almost all 5-HT-IR myenteric neurons in the gilthead sea bream proximal and distal intestine were also calbindin positive [25]. In the mammalian ENS, calbindin is considered a marker for sensory primary afferent neurons intrinsic to the intestine wall [59]. However, in fish gut, a clear-cut description of sensitive neurotransmitter pathways have not yet been provided. The possibility that 5-HT/CB-IR myenteric neurons may represent sensory neurons intrinsic to the fish myenteric plexus has been excluded, up to date, mainly on the base of morphological evidences. However, functional and electrophysiological studies are needed to validate such hypothesis [25].

### Calcitonin gene-related peptide

Calcitonin gene-related peptide (CGRP) is expressed by both neurons and enteroendocrine cells of fish gut and displays a wide range of actions including the control of peristalsis, vasodilation and regulation of food intake [60–62]. In the myenteric plexus of both proximal and distal *S*.*aurata* intestine, neurons expressing CGRP immunoreactivity represented the 27–29% of the total neuronal population. CGRP-IR neurons have been detected both in cross section of juvenile pejerrey (*Odontestes bonariensis*) proximal and distal intestine and in whole mounts of the Atlantic cod proximal and mid-intestine [15,60]. However, to the best of our knowledge, there are no data in the literature reporting the percentage of CGRP-IR myenteric neurons in other fish species. Our data are in line with the percentage of CGRP-expressing neurons observed in small mammals, such as mouse [45]. In the mammalian myenteric plexus, CGRP is considered as a sensory transmitter, expressed by intrinsic primary neurons, which project to ascending and descending interneurons involved in the control of peristalsis, as well as by fibers of extrinsic origin [4,45]. Electrophysiological and functional studies are compelling to demonstrate the existence of CGRP-containing intrinsic primary sensory neurons also in the gilthead sea bream myenteric plexus. However, the presence of multipolar neurons in both the proximal and distal intestine and the high degree of co-staining of CGRP-IR neurons with calbindin would suggest that CGRP might represent a putative neurotransmitter for intrinsic primary neurons also in this species. Functional and morphological studies carried out in the gut of Atlantic cod (*G*. *morhua*) and rainbow trout (*O*.*mykiss*) have demonstrated that CGRP represents an inhibitory neurotransmitter to intestinal smooth muscle. This action is exerted either directly on the target tissue or by modulating ascending and descending inhibitory pathways [15,63]. In gilthead sea bream intestine, these latter projections may be represented by the network of CGRP-IR varicose fibers running along the longitudinal muscle.

### Acetylcholine

The presence of a cholinergic innervation in gilthead sea bream intestine has been investigated using a commercial antibody raised against acetylcholine synthesizing enzyme, choline acetyl transferase (ChAT). The percentage of myenteric neurons staining for ChAT was different in the two intestinal regions studied, resulting around 16% of the total neuron number in the proximal intestine and 24% in the distal intestine. To the best of our knowledge, our data are the first ones expressing the percentage of cholinergic neurons in the teleost intestine. One of the major drawbacks, found by previous studies aiming to characterize cholinergic neurons in fish gut, was to satisfactorily label myenteric neurons with the available primary antibodies. In cross-sections of the Atlantic cod intestine, immunoreactivity to ChAT and to the vesicular acetylcholine transporter (VChAT), which specifically recognizes cholinergic terminals, revealed the presence of a few number of weakly stained myenteric neurons [64]. In contrast, high levels of ChAT activity were found in the gut of different freshwater teleosts, suggesting the presence of a consistent cholinergic innervation [8]. In our study, specific ChAT staining could be observed both in neuronal soma and in fibers running along the longitudinal layer of intestinal whole mount preparations, which are considered more suitable than cross sections for ChAT-IR myenteric neuron counting [65,66]. The number of ChAT immunopositive myenteric neurons in both regions of gilthead sea bream intestine were lower than those found in the ENS of most mammalian species, where the majority of enteric neurons are ChAT-IR [65]. This evidence further supports the existence of differences between the mammalian and fish ENS, that, however, needs to be confirmed by further studies on other fish species. ACh is an excitatory neurotransmitter released from excitatory motor neurons impinging on the smooth muscle of fish gut [55,58]. In our animal model, cholinergic excitatory motor neurons may be represented by the population of medium sized neurons with irregular shape and lamellar dendrites and by small uniaxonal oval neurons, innervating the circular and longitudinal smooth muscle coats, respectively, as observed in other species [65,66]. In agreement with data obtained in rat intestine myenteric plexus, ChAT immunoreactive neurons were larger in the proximal gilthead sea bream intestine than in the distal region [42,43]. However, the number of ChAT-IR neurons was significantly higher in the distal intestine. This observation well relates with the higher thickness of the smooth muscle layer, with respect to the proximal region.

In *S*. *aurata* intestine, the longitudinal muscle layer is innervated by numerous ChAT immunoreactive fibes, possibly generating from cholinergic interneurons and/or sensory neurons [65,67]. These latter may be represented by the subset of large ChAT-IR neurons with smooth cell bodies.

### Substance P

In the proximal and distal gilthead sea bream intestine SP-IR was found in scattered myenteric neurons and in trunks of varicose fibers along the longitudinal muscle axis. In the proximal and distal intestine, the percentage of SP-IR myenteric neurons was 18% and 28%, respectively. Several reports are available on the role of tachykinins in fish gut, however, ours is the first report to show the percentage of SP-containing neurons in a fish species. Our data are in good agreement with the percentage of SP-IR neurons observed in small mammal intestine, such as the bank vole (*Myodes glareolus*) and the mouse [43,68]. In analogy with the mammalian ENS, in the most phylogenetically advanced fish species, such as teleosts, SP may influence intestinal motility acting as a co-transmitter in cholinergic excitatory neurons to the smooth muscle layers [9,48]. In the Atlantic cod and rainbow trout, SP exerts both a direct excitatory role on the gut smooth muscle cells and an indirect effect, by activating both cholinergic and serotoninergic neurons [9, 57, 69]. In the gilthead sea bream intestine, we prevalently observed small to medium sized uniaxonal neurons either with a round soma or short dendrites or with an oval shape, which may represent excitatory motor neurons to the circular and longitudinal smooth muscle layer, respectively. The average size of SP-IR neurons was higher in the proximal intestine than in the distal intestine, as observed for the two other main transmitters present in enteric motor neurons, NO and ACh. Consistent with the concept that SP is a co-transmitter in cholinergic neurons, the percentage of SP expressing neurons in the distal intestine was significantly higher than in the proximal region, as demonstrated also for ACh containing neurons. In agreement with other studies, we observed an intense SP staining in trunks of fibers running along the longitudinal muscle layer, which may represent fibers emerging from interneurons with a prevalent oral projection, as demonstrated in the Atlantic cod intestine [64].

## Conclusions

In this study, we give a first description of myenteric plexus chemical coding in the juvenile gilthead sea bream (*Sparus aurata*) proximal and distal intestine. The main excitatory and inhibitory neurochemicals of fish intestinal muscular coat, namely ACh, SP, 5HT, and NO, VIP, CGRP, respectively, are present in our animal model. In addition, some populations of neurons with characteristics that potentially fulfill the criteria of ascending and descending interneurons as well as putative sensory neurons were identified. In the myenteric plexus of the proximal and distal intestine, the expression of the main neurotransmitter pathways was largely superimposable, although some differences emerged, which may be related to morphological peculiarities of each region. The most important are the increased density of myenteric neurons and soma size in the proximal intestine, which may depend on the major volume and thickness of the tissue innervated. In contrast, neurons containing acetylcholine and SP were more abundant in the distal intestine, probably owing to the higher thickness of the smooth muscle layer.

The data obtained in the present study may contribute to expand the knowledge on the intrinsic innervation regulating the gut function in gilthead sea bream, which is one of the most farmed teleost species in the Mediterranean area.
